# Impact of Bone Grafts Containing Metformin on Implant Surface Hydrophilicity: An In Vitro Study

**DOI:** 10.3390/dj13120611

**Published:** 2025-12-18

**Authors:** Rahul Minesh Shah, Nina Anderson, Rafael Delgado-Ruiz, Georgios Romanos

**Affiliations:** 1 Department of Periodontics and Endodontics, School of Dental Medicine, Stony Brook University, Stony Brook, NY 11794, USA; rahul.shah@stonybrookmedicine.edu; 2 Department of Oral Biology & Pathology, School of Dental Medicine, Stony Brook University, Stony Brook, NY 11794, USA; nina.anderson@stonybrookmedicine.edu; 3 Department of Prosthodontics and Digital Technology, School of Dental Medicine, Stony Brook University, Stony Brook, NY 11794, USA; rafael.delgado-ruiz@stonybrookmedicine.edu

**Keywords:** bone grafting, drug delivery, hydrophilicity, implant surface, metformin

## Abstract

**Background/Objectives**: The effect of metformin combined with bone grafting materials and its effect on the hydrophilicity of different implant surfaces has not been investigated. Investigation of the use of metformin as a therapeutic for implant surface treatment may be useful in improving overall implant longevity and success. **Methods**: Herein, a 1.5% metformin solution was created with crystalline metformin and distilled water. Titanium alloy (machined surface), titanium with sandblasted, large-grit acid-etched surface (Ti-SLA), and zirconia (SDS) surfaces were treated with five different solutions: 0.9% sodium chloride (Group A), bovine cancellous bone graft (Bio-Oss^®^)/0.9% sodium chloride solution (Group B), Bio-Oss^®^ bone graft with metformin/0.9% sodium chloride solution (Group C), algae-based bone graft (AlgOss^®^)/0.9% sodium chloride solution (Group D), and AlgOss^®^ bone graft with metformin/0.9% sodium chloride solution (Group E). Hydrophilicity tests utilizing droplet angle measurements (n = 20 droplets/disk) of each of the solutions were carried out (total N = 600 contact angle measurements). Statistical comparison between treatment groups for each implant surface using ANOVA and Bonferroni correction at *p* < 0.05 was performed. **Results**: Analyses revealed a statistically significant improvement in hydrophilicity for group C compared to group B (*p* < 0.05) in Ti-alloy, but a significant decrease in hydrophilicity for group E compared to group D in Ti-SLA. Zirconia surfaces displayed a decrease in hydrophilicity for all groups compared to group A. **Conclusions**: Thus, there were varying effects of combined metformin and bone graft on implants.

## 1. Introduction

Dental implant materials and their surface characteristics remain a considerable area of research for improving overall prognosis and implant survival. Commercially pure titanium, titanium alloys, and zirconia are among the most used materials for dental implants. Their surfaces are specifically modified to enhance biocompatibility, mechanical durability, and integration within the patient’s biological environment [1,2,3]. A major predictor for implant biocompatibility is hydrophilicity. Increased hydrophilicity has been shown to correlate with cell adhesion and proliferation directly, disbarring the effects of protein adsorption [4]. Thus, hydrophilicity has been shown to significantly impact an implant’s ability to promote osseointegration and tissue regeneration at the implant site [5]. Modifications to implant surfaces to increase hydrophilicity may improve long-term outcomes of the implant [5,6].

Systemic risk factors, such as diabetes mellitus, may increase the risk of developing peri-implantitis. In settings of uncontrolled diabetes mellitus, there may be an increase in marginal bone loss and pocket depth around their implants [7,8,9,10]. However, combating diabetes mellitus using glucose-controlling drugs, such as metformin, has been shown to lower HbA1c levels, accelerate wound healing, and improve physiological outcomes [8,11]. Its effects on clinical attachment loss in the periodontium have been noted to have positive results, leading to its use as an adjunctive medication in periodontal disease. In clinical trials and in vitro studies, metformin was incorporated using a topical application gel for deep periodontal pockets [12]. In these studies, the standard dosage for in vivo therapeutic metformin delivery has been set at 0.5–1.5% metformin, where more favorable results were found with higher concentrations of metformin [13]. The promising results presented in these studies and the hydrophilic nature of metformin give rise to the possibility of its use for implant surface treatment.

Using bone grafts to scaffold and facilitate bone formation in alveolar ridge defects has enabled dental implants to remain a viable treatment option for most patients [14,15,16]. Bone grafts are crucial for socket preservation, guided bone regeneration, and bone augmentation in alveolar ridges with reduced dimensions. Its use may be indicated before implant placement, during implant placement, or in the treatment of implant bony defects. Given the clinical importance of bone grafts in implantology, their surface interactions with implants must be considered. These surface interactions may exhibit varying effects on the physical characteristics of the implant interface and, in turn, the biological environment [17]. As a major predictor for implant biocompatibility, the hydrophilicity of an implant surface can be used to select implant surfaces that may respond better to the environment in which they are placed. Newman et al. [17] examined the effect of liquid supernatants after using two bovine-based bone grafting solutions with saline on different implant surfaces. It was found that bovine grafting materials may have a statistically significant negative impact on the hydrophilicity of zirconia surfaces compared to titanium surfaces. This demonstrates that the use of specific grafting materials for peri-implant defect therapy might poorly affect the hydrophilicity of the implant surface. Consequently, systemic conditions and chemical interactions with exogenous materials, such as bone grafts, can alter the implant’s hydrophilicity, eliciting unfavorable outcomes [17]. Investigation of mechanisms or therapeutics to improve hydrophilicity is crucial to establishing a favorable implant prognosis when bone grafts are used.

This in vitro study aimed to evaluate the effects of localized therapeutic metformin in combination with algae and bovine-based bone graft materials on the hydrophilicity of implant surfaces. The null hypothesis tested was that metformin, when used in conjunction with either the algae or bovine-based grafting solutions, does not alter the hydrophilicity of titanium and zirconia disk surfaces.

## 2. Materials and Methods

The three different dental implant surfaces that were used came in the form of small, non-contaminated circular disks and were:

1. Ti-alloy–Machined disk (myplant GmbH, Neuss, Germany);
2. Ti-sandblasted and acid-etched surface (SLA) disk–(myplant GmbH, Neuss, Germany);
3. Zirconia (SDS^®^, Swiss Dental Solutions, Kreuzlingen, Germany)

The evaluation of metformin and its effect on hydrophilicity for each surface was done using four different wetting solutions for each surface:

Group A: 0.9% sodium chloride (control).

Group B: Supernatant of the mixture of bovine, deproteinized spongious bone substitute (Bio-Oss^®^ S, cancellous, granules 0.25–1 mm, Geistlich, Wolhusen, Switzerland) with 0.9% sodium chloride solution.

Group C: Supernatant of the mixture of Bio-Oss^®^ S cancellous bone graft with 1.5% metformin solution (Enzo Life Sciences, Farmingdale, NY, USA, 11735l 270-432-G005) in 0.9% sodium chloride.

Group D: Supernatant of the mixture of marine 20% hydroxyapatite/80% β-tricalcium phosphate red algae-based material (AlgOss^®^, myplant GmbH, Neuss, Germany) and 0.9% sodium chloride solution.

Group E: Supernatant of the mixture of AlgOss^®^ bone graft with 1.5% metformin solution in 0.9% sodium chloride solution.

These five groups were based on the inclusion of two different types of xenograft bone grafts that are used in clinical practice. Testing the bone graft’s effect on hydrophilicity independently prior to inclusion of metformin was important for standardization.

The 1.5% metformin solutions were created using distilled water and crystalline metformin. Groups B–D used either a bovine deproteinized mineral or an algae-based bone graft. As per clinical protocols, in treatment groups B–D, 0.5 g of each bone grafting material was placed in a test tube and mixed thoroughly for 1–2 min in 0.9% sodium chloride at room temperature. Through the hydration of the bone grafts, particles could adhere to each other, leaving a supernatant consisting of grafting particles and saline solution. For groups C and E, an equal mixture of 1.5% metformin solution and aspirated bone grafting supernatant was mixed to create a stock solution for the hydrophilic solutions. For groups B and D, the supernatant of sodium chloride was aspirated directly for hydrophilicity testing.

To examine metformin’s effect in conjunction with bone grafting materials, the sessile drop technique was used to determine the effect each solution had on each dental implant surface. A contact angle goniometer (Ossila^®^, Sheffield, UK) was used to obtain the contact angles of each wetting solution for the three dental implant surfaces. The goniometer was attuned, and each disk was cleaned and dried at room temperature and placed on the stage. A calibrated micropipette (GILSON pipetman, Fisher scientific, Hampton, NH, USA) was used to place 10 µL of each wetting solution at the center of each implant surface disk. Using the Ossila^®^ video recording software (Ossila. v3.0.6.0 software), the process of dispensing the droplet could be recorded and analyzed. Fifteen experimental groups, each composed of twenty repetitions of droplets and two obtained contact angles (N = 600 contact angle measurements), resulted from this. Disks were cleaned and dried following each droplet, and a new implant surface disk was used for each experimental solution. A contact angle (θ) is obtained where θ > 90° is considered hydrophobic and θ < 90° is considered hydrophilic [18]. Given that each experimental solution used one disk, five different implant disks were used for each given surface. A total of 15 disks were used throughout the study (5 Ti-alloy, 5 Zirconia, 5 Ti-SLA).

**Figure 1:**
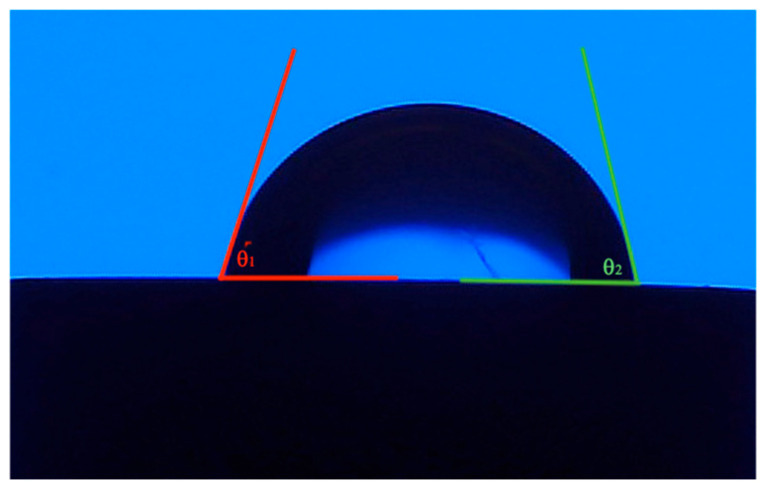
Visual representation of measured contact angles θ_1_ and θ_2_ between implant surface and wetting solution using Ossila^®^ software and goniometer.

The Ossila^®^ goniometer has an implemented software that uses edge detection, allowing for differentiation between the dental implant surface and the droplet edge. Using this software, an image was captured one second following droplet administration to each surface, and a left and right contact angle was obtained. This angle obtained was the combination of two lines: one line that extended across the sample implant surface and another line that was the tangent of the droplet from the intersection of the implant surface and approximated droplet edge (Figure 1).

### Statistical Analysis

A power analysis was conducted to determine the minimum sample size required to test the study’s hypothesis. The a priori power analysis showed that to achieve 80% power for detecting a medium/large effect at a significance level of 0.05, n = 20 droplets per experimental group per disk was required. Using G*Power 3.1, a post hoc power analysis indicated that the study had 99% power to detect group differences. Prior to conducting the ANOVA, data were examined for normality using the Shapiro–Wilk test, and Homogeneity of variances was assessed using Levene’s test. Assumptions were satisfied for medium to large effect with an η^2^ of 0.753 in the Ti-alloy group, 0.620 for the Ti-SLA group, and 0.435 for the zirconia group. There were five experimental groups for each type of implant surface disk. One implant disk was used to test each experimental solution group. Statistical comparison tests between experimental groups were performed using one-way ANOVA with variance and Bonferroni post hoc multiple comparison test to determine significant mean differences at the 0.05 level. It is important to note that the implant surface disks were reused for each experimental group. Disks were dried and cleaned following droplet placement and only replaced when testing new experimental groups.

## 3. Results

*The Ti-alloy machined surfaces* resulted in mean wetting angles of 91.4° ± 3.67 for group A, 90.1° ± 3.27 for group B, 83.8° ± 2.55 for group C, 79.9° ± 3.94 for group D, and 77.7° ± 1.98 for group E (Table 1). When conducting the Bonferroni correction (Table 2), a statistically significant mean difference (*p* < 0.05) was found between groups A and B–E, indicating that all treatment wetting solutions caused a significant increase in hydrophilicity. Additionally, a statistically significant increase in hydrophilicity was found between group C and group B, as well as group E and group D, indicating that the addition of metformin in the bone grafting solutions significantly increased the hydrophilicity of the titanium alloy surfaces. In terms of gross comparison, the algae-based bone graft and metformin together had a more significant increase in hydrophilicity compared to the use of bovine-based bone graft with metformin.

*The Ti-SLA surfaces* resulted in mean wetting angles of 91.4° ± 2.63 for group A, 84.9° ± 3.43 for group B, 82.4° ± 2.49 for group C, 81.3° ± 2.72 for group D, and 86.3° ± 3.06 for group E (Table 1). When conducting the Bonferroni correction (Table 3), a statistically significant difference was found between groups A and B–E, indicating that all treatment wetting solutions caused a significant increase in hydrophilicity. A statistically significant increase in hydrophilicity was found between group B and group C (*p* < 0.05). A statistically significant decrease in hydrophilicity was found between group D and group E, indicating that the addition of metformin in the bovine-based bone grafting solution only increased (*p* < 0.05) the hydrophilicity of the Ti-SLA dental implant surface significantly. In contrast, using algae-based bone graft and metformin significantly decreased the hydrophilicity of the Ti-SLA disk surfaces. There was still a significant increase (*p* < 0.05) in hydrophilicity in all treatment groups compared to the use of saline alone.

**Figure 2:**
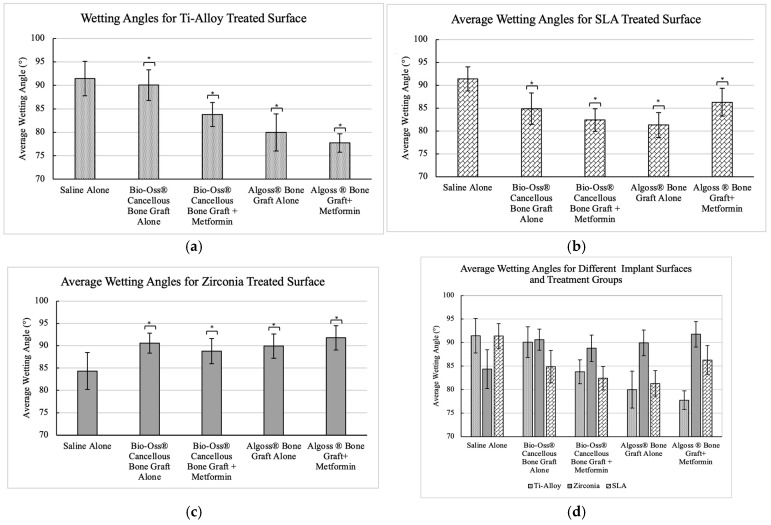
(**a**) Visual comparison of mean contact angles and standard deviations for Ti-alloy when treated with different wetting solutions (* denotes where *p* < 0.05 when compared to saline alone) (**b**) Visual comparison of mean contact angles and standard deviations for Ti-SLA surfaces when treated with different wetting solutions (* denotes where *p* < 0.05 when compared to saline alone) (**c**) Visual comparison of mean contact angles and standard deviations for zirconia surfaces, when treated with different wetting solutions (* denotes where *p* < 0.05 when compared to saline alone). (**d**) Visual comparison of mean contact angles and standard deviations for Ti-alloy, Ti-SLA, and zirconia surfaces when treated with different wetting solutions.

*The zirconia surfaces* resulted in wetting angles of 84.3° ± 4.13 for group A, 90.6° ± 2.24 for group B, 88.8° ± 2.83 for group C, 89.9° ± 2.73 for group D, and 91.8° ± 2.70 for group E (Table 1). Zirconia surfaces seemed to display opposite results when compared to the Ti-alloy surfaces. When conducting the Bonferroni correction (Table 4), a statistically significant mean difference was found between groups A and B–E (*p* < 0.05). In this case, all treatment wetting solutions caused a significant decrease in hydrophilicity. There was a statistically significant decrease in hydrophilicity when metformin was used with the algae-based bone graft, and no significant change in hydrophilicity when metformin was used with the bovine-mineral bone graft. Gross visual comparison of mean wetting angles and their standard deviations can be seen in Figure 2.

## 4. Discussion

This study aimed to measure (a) the effect of using bovine-based bone graft and algae-based bone graft on the hydrophilicity of the implant surfaces, (b) the effect of metformin within hydrated bone grafts on the hydrophilicity of implant surfaces, and (c) if there was any difference in hydrophilicity of implant surfaces between the two hydrated bone grafts when used with metformin. The null hypothesis can be rejected for the Titanium-alloy machined surfaces and the Ti-SLA surfaces when bovine mineral grafting material was used. Zirconia smooth surfaces presented results that failed to reject the null hypothesis.

Certain grafting materials may inhibit cell proliferation due to potential toxicity. For example, a previous study showed that bovine minerals, mineralized cancellous human bone, and hydroxyapatite caused a toxic effect on primary osteosarcoma osteoblast-like SaOS-2 cells [19]. Despite bovine grafting materials having potential reported cytotoxic effects, their impact on the mechanical characteristics of dental implant surfaces yielded favorable mechanical results with increases in hydrophilicity for specific surfaces [18].

For the Ti-alloy machined surfaces, there was a statistically significant increase in hydrophilicity for both bone grafting solutions using metformin compared to using bone graft solutions alone. Studies conducted by Newman et al. [17] have shown that bone grafting solutions have increased the hydrophilicity of titanium-based implant surfaces, which supports the results found in the present study. The further increase in hydrophilicity due to the influence of metformin may be attributed to the hydrophilic nature of the drug and the chemical interaction between the bone graft, implant surface, and drug [19]. A significant increase in hydrophilicity was noted when algae-derived bone grafts were hydrated and mixed with metformin compared to when bovine mineral and metformin were used together. This indicates that metformin has a more positive effect on surface hydrophilicity when used with algae-based bone graft. AlgOss^®^ contains 20% hydroxyapatite and 80% β-tricalcium phosphate, both of which are hydrophilic [20,21]. This may have enhanced the hydrophilicity of the solution when the algae-based bone graft was combined with metformin, giving rise to the favorable surface hydrophilicity.

For the Ti-SLA surfaces, there was a significant change in hydrophilicity for both bone grafting solutions using metformin when compared to the use of bone graft solutions alone. Using the algae-based bone graft with metformin significantly decreased hydrophilicity, whereas using the bovine-based bone graft and metformin increased hydrophilicity. It is important to note that all experimental groups resulted in a statistically significant increase in hydrophilicity, indicating that the algae-based bone graft was not the reason for the decrease in hydrophilicity. Rather, the metformin was the reason. This is deduced due to the algae-based bone graft solution alone being more hydrophilic than the combination of the algae-based bone graft and metformin. It should be noted that studies comparing different grafting materials to each other have revealed differences in the hydrophilicity of implant surfaces after hydration of the grafting materials [17]. This variance in hydrophilicity may also be associated with the use of metformin and its interaction with the bone graft and the altered porous surface. Further research needs to be conducted with a focus on examining the chemical interaction between a sandblasted acid-etched surface, metformin, and the chemical components of AlgOss^®^ bone graft to explain the results here.

For the zirconia surfaces, there was a statistically significant decrease in hydrophilicity for all wetting solutions compared to the saline control. This indicates that the use of both types of bone graft has adverse effects regarding implant surface hydrophilicity. Although studies have shown that combining metformin with scaffolds, such as bone grafts, has led to improvement in bone regeneration in bone defect models, its effects on implant surfaces should also be considered [22]. Its importance for integration in the biological environment cannot be understated. The data here support the findings of previous studies where the inclusion of bone grafting material, specifically bovine mineral grafts, decreased the hydrophilicity on zirconia surfaces [17]. Characteristics should be investigated, and how the oxide surface of Zirconium interacts with bone grafts and the surrounding biological environment. The differing results found for zirconia compared to the titanium surfaces must be further analyzed on a chemical and mechanical level to ascertain the rationale for the unfavorable results. Metformin failed to significantly increase the hydrophilicity of the zirconia implant surface in this study, indicating it may not be beneficial for zirconia surfaces.

These findings may provide preliminary insights for clinicians who have patients with periodontal intrabony or peri-implantitis defects. Surface modifications, such as increasing hydrophilicity of the implant surface, may have a positive impact on clinical results and outcomes. This is supported by previous in vivo and in vitro studies where increases in hydrophilicity of implant surfaces led to better prognosis of the implant and osseointegration [1,2,3]. The simulation of metformin local delivery to implant sites directly along with bone grafting materials showed that hydrophilicity characteristics may be favorably altered such that the long-term functionality of the implant is improved. Another point of interest that should be considered is the effect of systemic metformin administration and local therapeutic metformin on implant surfaces in vivo. This gives rise to the need for further in vivo validation before clinical application of this method. This will ensure there are no biological contraindications and that the results in this study are accurate.

Limitations of this study include its narrow scope in terms of the type of analysis done on dental implant surfaces treated with the experimental wetting solutions. Replication of the study can be done with multiple disks and by a different examiner to reduce operational bias and increase the strength of the findings. Surface characteristics, such as AFM roughness and topography, could be examined to achieve a more holistic surface change pattern. The residual roughness of the dental implant surfaces could be analyzed to determine if the wetting solutions led to any topographical changes, as an increased roughness would allow for better bone integration with the implant. This can be done through surface electron microscopy to quantify the surface roughness of each implant surface following treatment with each wetting solution [23]. In vivo studies in animal models should also be carried out to gain a better understanding of the biological effects of the therapy, both systemically and locally at the site of delivery. These studies should look at cell adhesion, proliferation, and protein adsorption resulting from the therapeutic regimens presented here.

## 5. Conclusions

Within the limitations of this experimental in vitro study, it can be concluded that metformin combined with algae-based bone graft supernatant improves the hydrophilicity of Ti-alloys. Metformin combined with bovine-based bone graft improves the hydrophilicity of Ti-SLA surfaces. However, metformin does not alter the hydrophilicity of zirconia surfaces. This indicates that the use of metformin with bone graft products may have varying effects on different implant surface characteristics. These results may be pertinent to future research for in vivo applications of metformin in promoting implant hydrophilicity and osseointegration.

## Figures and Tables

**Table 1:** Mean, standard deviation, and 95% confidence intervals for contact angles for each wetting solution and Ti-alloy, Ti-SLA, and zirconia surfaces.

Dental Implant Surface	Wetting Solution	N	Mean (°)	Std. Dev	95% CI
Ti-Alloy	Group A	40	91.4	3.67	[87.73, 95.07]
	Group B	40	90.1	3.27	[86.83, 93.37]
	Group C	40	83.8	2.55	[81.25, 86.35]
	Group D	40	79.9	3.94	[75.96, 83.84]
	Group E	40	77.7	1.98	[75.72, 79.68]
Ti-SLA	Group A	40	91.4	2.63	[88.77, 94.03]
	Group B	40	84.9	3.43	[81.47, 88.33]
	Group C	40	82.4	2.49	[89.91, 84.89]
	Group D	40	81.3	2.72	[78.58, 84.02]
	Group E	40	86.3	3.06	[83.24, 89.36]
Zirconia	Group A	40	84.3	4.13	[80.17, 88.43]
	Group B	40	90.6	2.24	[88.36, 92.84]
	Group C	40	88.8	2.83	[85.97, 91.63]
	Group D	40	89.9	2.73	[87.17, 92.63]
	Group E	40	91.8	2.70	[89.10, 94.50]

**Table 2:** Bonferroni correction to compare wetting solutions at the *p* < 0.05 level (denoted by *) was conducted for the Ti-alloy surfaces. A statistically significant mean difference was found between group A and groups B–E, with all differences being a decrease in contact angle compared to Group A. A statistically significant increase in hydrophilicity was found between group C and group B, as well as between group E and group D.

(I) Group	(J) Group	Mean Diff. (I–J)	Std. Error	Sig.	95% CI
Group A	Group B	1.35550	1.00074	1.000	[−1.5207, 4.2317]
	Group C	7.65850	1.00074	0.000 *	[4.7823, 10.5347]
	Group D	11.45900	1.00074	0.000 *	[8.5828, 14.3352]
	Group E	13.71300	1.00074	0.000 *	[10.8368, 16.5892]
Group B	Group A	−1.35550	1.00074	1.000	[−4.2317, 1.5207]
	Group C	6.30300	1.00074	0.000 *	[3.4268, 9.1792]
	Group D	10.10350	1.00074	0.000 *	[7.2273, 12.9797]
	Group E	12.35750	1.00074	0.000 *	[9.4813, 15.2337]
Group C	Group A	−7.65850	1.00074	0.000 *	[−10.5347, −4.7823]
	Group B	−6.30300	1.00074	0.000 *	[−9.1792, −3.4268]
	Group D	3.80050	1.00074	0.003 *	[0.9243, 6.6767]
	Group E	6.05450	1.00074	0.000 *	[3.1783, 8.9307]
Group D	Group A	−11.45900	1.00074	0.000 *	[14.3352, −8.5828]
	Group B	−10.10350	1.00074	0.000 *	[−12.9797, −7.2273]
	Group C	−3.80050	1.00074	0.003 *	[−6.6767, −0.9243]
	Group E	2.25400	1.00074	0.266	[−0.6222, 5.1302]
Group E	Group A	−13.71300	1.00074	0.000 *	[−16.5892, −10.8368]
	Group B	−12.35750	1.00074	0.000 *	[−15.2337, −9.4813]
	Group C	−6.05450	1.00074	0.000 *	[−8.9307, −3.1783]
	Group D	−2.25400	1.00074	0.266	[−5.1302, 0.6222]

**Table 3:** Bonferroni correction to compare wetting solutions at the *p* < 0.05 level was conducted for the Ti-SLA surfaces. A statistically significant mean difference was found between group A and groups B–E, with all differences being a decrease in contact angle compared to Group A. A statistically significant increase in hydrophilicity was found between group B and group C, but a statistically significant decrease in hydrophilicity was found between group D and group E. *: statistically significant.

(I) Group	(J) Group	Mean Diff. (I–J)	Std. Error	Sig.	95% CI
Group A	Group B	6.52900	0.91222	0.000 *	[3.9072, 9.1508]
	Group C	9.00950	0.91222	0.000 *	[6.3877, 11.6313]
	Group D	10.0985	0.91222	0.000 *	[7.4767, 12.7203]
	Group E	5.11400	0.91222	0.000 *	[2.4922, 7.7358]
Group B	Group A	−6.52900	0.91222	0.000 *	[−9.1508, −3.9072]
	Group C	2.48050	0.91222	0.078	[−0.1413, 5.1023]
	Group D	3.56950	0.91222	0.002 *	[0.9477, 6.1913]
	Group E	−1.41500	0.91222	1.000	[−4.0368, 1.2068]
Group C	Group A	−9.00950	0.91222	0.000 *	[−11.6313, −6.3877]
	Group B	−2.48050	0.91222	0.078	[−5.1023, 0.1413]
	Group D	1.08900	0.91222	1.000	[−1.5328, 3.7108]
	Group E	−3.89550	0.91222	0.000 *	[−6.5173, −1.2737]
Group D	Group A	−10.09850	0.91222	0.000 *	[−12.7203, −7.4767]
	Group B	−3.56950	0.91222	0.002 *	[−6.1913, −0.9477]
	Group C	−1.08900	0.91222	1.000	[−3.7108, 1.5328]
	Group E	−4.98450	0.91222	0.000 *	[−7.6063, −2.3627]
Group E	Group A	−5.11400	0.91222	0.000 *	[−7.7358, −2.4922]
	Group B	1.41500	0.91222	1.000	[−1.2068, 4.0368]
	Group C	3.89550	0.91222	0.000 *	[1.2737, 6.5173]
	Group D	4.98450	0.91222	0.000 *	[2.3627, 7.6063]

**Table 4:** Bonferroni correction to compare wetting solutions at the *p* < 0.05 level was conducted for the zirconia surfaces. A statistically significant mean difference was found between group A and groups B–E, but all treatment wetting solutions caused a significant decrease in hydrophilicity. A statistically significant decrease in hydrophilicity for group E compared to group D, and no significant change in hydrophilicity for group C compared to group B. *: statistically significant.

(I) Group	(J) Group	Mean Diff. (I–J)	Std. Error	Sig	95% CI
Group A	Group B	−6.26200	0.94645	0.000 *	[−8.9822, −3.5418]
	Group C	−4.43650	0.94645	0.000 *	[−7.1567, −1.7163]
	Group D	−5.58200	0.94645	0.000 *	[−8.3022, −2.8618]
	Group E	−7.42300	0.94645	0.000 *	[−10.1432, −4.7028]
Group B	Group A	6.26200	0.94645	0.000 *	[3.5418, 8.9822]
	Group C	1.82550	0.94645	0.567	[−0.8947, 4.5457]
	Group D	0.68000	0.94645	1.000	[−2.0402, 3.4002]
	Group E	−1.16100	0.94645	1.000	[−3.8812, 1.5592]
Group C	Group A	4.43650	0.94645	0.000 *	[1.7163, 7.1567]
	Group B	−1.82550	0.94645	0.567	[−4.5457, 0.8947]
	Group D	−1.14550	0.94645	1.000	[−3.8657, 1.5747]
	Group E	−2.98650	0.94645	0.021 *	[−5.7067, −0.2663]
Group D	Group A	5.58200	0.94645	0.000 *	[2.8618, 8.3022]
	Group B	−0.68000	0.94645	1.000	[−3.4002, 2.0402]
	Group C	1.14550	0.94645	1.000	[−1.5747, 3.8657]
	Group E	−1.84100	0.94645	0.547	[−4.5612, 0.8792]
Group E	Group A	7.42300	0.94645	0.000 *	[4.7028, 10.1432]
	Group B	1.16100	0.94645	1.000	[−1.5592, 3.8812]
	Group C	2.98650	0.94645	0.021 *	[0.2663, 5.7067]
	Group D	1.84100	0.94645	0.547	[−0.8792, 4.5612]

## Data Availability

The original contributions presented in this study are included in the article. Further inquiries can be directed to the corresponding authors.
